# 16S rRNA amplicon sequencing characterization of caecal microbiome composition of broilers and free-range slow-growing chickens throughout their productive lifespan

**DOI:** 10.1038/s41598-019-39323-x

**Published:** 2019-02-21

**Authors:** Medelin Ocejo, Beatriz Oporto, Ana Hurtado

**Affiliations:** NEIKER-Instituto Vasco de Investigación y Desarrollo Agrario, Animal Health Department, Derio, 48160 Spain

## Abstract

Gut microbiota affects health, metabolism and immunity of the host, and in the case of livestock, also food-safety. Here, 16S rRNA gene high-throughput Illumina sequencing was used to describe the microbiome of chicken caeca in two different breeds and management systems throughout their whole productive lifespan. Broilers (Ross-308), as a fast-growing breed reared in an intensive system for 42-days, and a slow-growing breed of chicken (Sasso-T451A) reared in an extensive farming system with outdoor access for 86-days, were compared. The core microbiome and differentially abundant taxa, as well as taxa associated with age were identified. Age was identified as the strongest influencing factor in caecal microbiota composition, and, in general, each age-group showed an age-associated community profile, with a transition period at the middle of their lifespan. However, substantial differences were observed in the composition of caecal microbiota of both chicken breeds, microbiota being richer and more complex in free-range chicken than in broilers. Several taxa positively/negatively correlated with *Campylobacter* relative abundance were also identified. Especially noteworthy was the identification by microbial community comparison of microbiota profiles suggestive of dysbiosis in several free-range chickens, probably associated to the typhlitis observed in the lumen of their caeca.

## Introduction

Gut microbiota is considered as an additional organ due to its vital importance on the physiological, metabolic, immunological, and digestion and nutritional uptake functions of the host^1^. Considering its implication in food safety and public health^2,3^, gut microbiota of food-producing animals has become a topic of interest and the subject of extensive studies. Among food-producing animals, poultry is the source of one of the most consumed meat worldwide. While conventionally raised poultry continues to dominate the EU poultry industry, there is an increasing demand for what is considered less intensive and more welfare-friendly management practices. In this regard, free-range production is an attribute highly appreciated among consumers who are looking for value-added food products. Free-range production of chicken meat normally requires slow-growing breeds and age at slaughter is normally not less than 12 weeks^4^. Fast-growing commercial hybrids are not suitable for these production systems, which are rather used for intensive broiler production, slaugther age varying between 5 and 7 weeks.

Defining what constitutes a healthy gut microbiota is essential to help designing strategies to modulate its composition, not only in the realm of improving host performance and therefore enhance production, but also to maintain optimal host health and control zoonotic agents^5^ that can contaminate food of animal origin thus posing a risk for consumers health. However, there is no clear definition of a healthy microbiota^6^. Still, higher microbial diversity is commonly associated with a healthier host status, as the lack of sufficient diversity or evenness in a bacterial community structure appears to diminish its ability to withstand perturbation. In this sense, reduced microbial diversity has been associated with different intestinal disease states^6,7^. Furthermore, disruption of the gut microbiota structure that results in the elimination of subsets of beneficial bacteria, often leads to pathogen overgrowth, in conjunction with significant loss of microbial diversity^8^. Besides, factors such as diet^9–11^, rearing conditions^12^, host genetics^13^ and age^9,14–18^ can have an effect on poultry gut microbiota diversity, composition, and community structure. In addition, different regions of the bowel also harbor different microorganisms, as detailed in several reviews^3,19,20^ and recent studies^16,21^. The chicken caecum hosts the largest quantity and diversity of microbes along the chicken gastrointestinal tract since a prolonged retention of digesta occurs, and it is the main site of bacterial fermentation^9,22^. Dietary interventions have been used to control gut health problems related to overgrowth of certain intestinal bacteria (dysbacteriosis) both in animals and humans, or to control zoonotic pathogens that are often found in the gut of commercially raised chickens, like *Campylobacter*, *Escherichia coli*, *Salmonella* or *Streptococcus*, as reviewed elsewhere^5,23^. In a previous study, we showed the beneficial effects on performance and intestinal integrity of feeding broilers with a diet supplemented with dry whey and coated calcium butyrate^24^. Since poultry is considered the principal reservoir of *Campylobacter* and main source of human campylobacteriosis^25^, in that study we also tested diet effects on *Campylobacter* colonization and dissemination but none of the tested diets provided the chicks any differential degree of protection against *Campylobacter* infection^24^. However, effects on microbiota community profiles were then not measured. With the onset of high-throughput sequencing technologies, communities as diverse and complex as gut microbiota, can be sequenced to unprecedented depth and coverage more accurately. Targeted sequencing, also defined as amplicon sequencing, is one of the main DNA-based approaches of this technology at the time of publication^26,27^. It uses the amplification of conserved regions such as 16S ribosomal RNA (rRNA) genes in the analysis of bacterial community profiling. Caecal microbial communities in chickens have been assessed in previous studies. However, most efforts have focused on broilers and studies in other breeds and management systems are scarce. Furthermore, not many studies fully covered the complete productive period^28–30^ or provided a deep sequencing coverage^15,28,31,32^.

The aim of this study was to thoroughly characterize the caecal microbiota in two breeds of chickens fed with different diets and bred under different production systems throughout their complete lifespan; Ross-308 broilers reared for 42 days, as the fast-growing breed used in intensive chicken meat production, and a slow-growing breed (Sasso-T451A) reared in a free-range system for 84 days. The effect of breed, diet and production system was assessed to obtain a comprehensive knowledge of how microbiota develops as chickens grow in the different phases of their productive life, and particularly the following questions were addressed: (i) How do caecal microbial communities change during the productive life of chickens? (ii) Does supplementation of diet with calcium butyrate and dry whey influence the caecal microbial community patterns in broilers? iii) Do caecal microbiome temporal dynamics vary between fast-growing and slow-growing breeds? iv) Which microbial taxa are representative of each age-group? (v) Is there a core microbiota for all age-groups in each breed, and a core microbiota for both breeds throughout their productive life? (vi) Which taxa tend to coexist with *Campylobacter* in the chicken caeca?

## Methods

### Subjects discription

#### Broiler chickens

Broiler (Ross-308) chickens were obtained in the frame of an experimental study carried out to assess the effect of dietary supplementation with dry whey powder (a prebiotic), coated calcium butyrate (a salt of a short-chain fatty acid - SCFA) or their combination, in terms of productive performance, duodenal histological integrity, and *Campylobacter* colonization and dissemination^24^. Briefly, 600 one-day-old Ross-308 chickens were placed into 20 ground pens (2.5 × 1 m each) and assigned to one of 4 dietary treatments (5 replicates of 30 chicks per treatment) following a randomized complete block design. The birds were fed two-phase corn/soybean-based diets during the starter (0–20d) and grower-finisher (21–42 d) periods, with different supplementations as follows: (1) basal diet with no supplementation as control (CO); (2) diet containing 6% dry whey powder (WH); (3) diet supplemented with coated calcium butyrate at 0.1% (BU) and 4) diet containing 6% whey and 0.1% calcium butyrate (SY). The diets were formulated to meet Ross-308 broiler requirements^33,34^ and to provide equal nutrient profiles. Chicks were reared for 42 days. Feed and water were provided *ad libitum*. To simulate a common situation in broiler production, 6 chickens per pen were experimentally inoculated with *Campylobacter jejuni* at 15 days of age, when natural infections are usually reported^35,36^, and the infection was monitored until the end of the productive period^24^.

#### Free-range slow-growing chickens (FRC)

Slow-growing meat chickens (Sasso-T451A) were provided by a local commercial free-range poultry farm. Chickens were reared for 12 weeks on a semi-extensive management system and slaughtered with average body weight of 2,235 kg. Feed and water were provided *ad libitum*. Feed consisted of grain, composed of at least 60% corn and different proportions of wheat and soya according to age requirements, as follows: 1–35d, 5% wheat and 30% soya; 36–61d, 11% wheat and 24% soya; and, 61–84d, 12% wheat and 23% soya. In addition, from day 26 birds had free daytime access to grassland yards and grass became part of their diet. The flock included 3,650 birds, and stocking density was 11 chickens/m^2^ within houses, and 2 m^2^ per chick outdoors. No antibiotics were administered, and the only vaccine given was to control coccidiosis. The all-in all-out system was implemented. The region where the farm was located has an Atlantic climate, and during the production period (24th May–16^th^ August 2016), mean temperature was 19.9 °C (mean maximum, 24.7 °C; mean minimum, 15.2 °C) and the total accumulated rainfall was 102.9 l/m^2^.

### Sample collection

Both breeds were sampled in different years but within the same period of the year. Broilers were sampled in May-June 2015 and FRC were sampled between May and August 2016. A total of 80 broilers (1 per pen, i.e., 5 replicates per diet) were randomly selected at four time points (3, 14, 29, and 42 days of age) and 38 FRC were sacrificed at five time points (4, 18, 39, 58 and 81 days of age). All chickens belonged to the same productive lot within the breed type and represented the average weight at each group. No feed withdrawal was performed before being euthanized by exposure to carbon dioxide (CO_2_). Necropsy was immediately performed, and caeca were aseptically dissected, and their contents collected for DNA extraction.

### Ethics statement

The protocols for the study carried out in broilers were approved by the local Animal Ethics Committee (Diputación Foral de Álava, Register no. 1821/12.05.2014). Sample collection in the free-range poultry farm was carried out by the veterinary clinician after obtaining informed oral consent from the farm owner. In both experiments, handling of the birds was carried out in compliance with European Community Directive 2010/63/EU and its transposition into national legislation through the Royal Decree 53/2013 on the protection of birds used for scientific purposes.

### DNA Isolation and next-generation sequencing (NGS)

Genomic DNA was extracted from approximately 0.15 g of caecal content using PowerSoil™ DNA Isolation Kit (MoBio Laboratories, Carlsbad, CA, USA) according to the manufacturer’s instructions. Whereas for broilers the content of both caeca was mixed before DNA purification, for FRC caecal content from each caecum was processed individually as replicates (except for the 4-day-old group due to limited sample availability). In order to prevent repetitive freeze-thawing, two DNA aliquots were prepared, one which was immediately stored at −80 °C until shipping to the sequencing service and another one to be used for any other analyses. Concentration of DNA was determined by spectrophotometry (Nanodrop ND1000; Thermo Scientific, USA) and its integrity was visually assessed by 0.8% agarose gel alectroforesis.

Normalized DNA was submitted to the Centre for Genomic Research of the University of Liverpool (UK) for PCR amplification, library preparation and 16S high-throughput sequencing. The V4 hypervariable region of the bacterial 16S rRNA genes present in each caecal DNA sample was amplified by PCR using the universal primer set 515F/806R according to the protocol described elsewhere^37^. A dual-indexing amplification and sequencing approach was used and the resulting amplicons were purified and subjected to paired-end sequencing (2 × 250 bp) of one pooled amplicon sample on two runs of the Illumina MiSeq platform, one for broilers and another for FRC.

### Bioinformatic Analysis | Sequence processing and data analysis

Illumina adapter sequences were trimmed from the raw fastq files using Cutadapt v1.2.1^38^ and reads below a window quality score of 20 were trimmed using Sickle v.1.200^39^. Reads shorter than 10 bp were discarded, primers were trimmed with Cutadapt in the paired-end mode and quality of the reads was assesed using FastQC v.0.11.5 quality-control tool (Babraham Bioinformatics, Cambridge, United Kingdom)^40^. Subsequent analyses were performed within R environment^41^ following DADA2 Pipeline (Tutorial (1.6) in https://benjjneb.github.io/dada2/tutorial.html) by adjusting parameters to our dataset. The end product was a non-chimeric amplicon sequence variants (ASVs) table^42^ which records the number of times each ASV (sequence differing by as little as one nucleotide) was observed in each sample. Taxonomy assignment of representative ASVs was performed with DADA2 against GreenGenes reference database (v.13.8)^43^ with the bootstrapping threshold set to 80%. ASVs composed by less than 10 sequences in all samples were filtered out.

### Ecological and Statistical analyses

Downstream analyses and graphical outputs were generated with different packages in R v.3.3.2^41^. Continuous variables were tested for normality with the Shapiro-Wilk test. Rarefaction curves were constructed on unnormalized ASV-level data using Vegan package^44^ for each age-group to assess sequencing effort. Phyloseq v.1.19.1^45^ was used to visualize abundance of microbial taxonomic composition, estimate biodiversity, create heatmaps and perform normalization and differential abundance tests. Both alpha and beta diversity metrics were used to estimate microbial communities diversity. Species richness (Observed ASVs and Chao1) and evenness (Shannon and Simpson index) were calculated for alpha diversity estimations. To compare alpha diversity metrics among groups, non-parametric Kruskal-Wallis test was conducted, followed by subsequent pairwise comparisons with Wilcoxon rank sum test adjusted using the Benjamini–Hochberg (B-H) method. Total sum scaling was used as normalization method to account for variability in the number of reads between samples. For beta diversity analysis, dissimilarity matrix between samples was calculated with Bray Curtis method, and it was further visualized with a Non-Metric Multidimensional Scaling (NMDS) ordination technique. Bray Curtis dissimilarity was also used to perform hierarchical clustering using hclust with the unweighted pair-group method with arithmetic mean (UPGMA) method. To study the effect of age and diet on microbiota composition variability between samples based on beta diversity distance matrices, we used permutational multivariate analysis of variance (PERMANOVA) with the adonis function from R’s Vegan package^44^. Pairwise PERMANOVA comparisons were done with a function written by Pedro Martinez Arbizu available in the ResearchGate questions section. (https://www.researchgate.net/post/How_can_I_do_PerMANOVA_pairwise_contrasts_in_R) also adjusted with B-H method. To test for homogeneity of multivariate dispersions (i.e. deviations from centroids) among age-groups or diets, permutation multivariate analysis of dispersion (PERMDISP) was conducted with the function betadisper and permutest also from Vegan package.

In an attempt to look for differentially abundant taxa among the different age-groups and diets, DESeq2 (v.1.14.1)^46^ was carried out by performing the Wald significance test with a parametric fit type and multiple-inference correction by B-H method. Linear Discriminant Analysis Effect Size (LEfSe) algorithm with LDA effect size threshold of 2 (on a log_10_ scale) was applied after agglomerating data to genus level for evidencing potential biomarkers linked to age and diet. The outputs of both approaches were combined to identify potential biomarkers as those significantly representative by LEfSe and differentially abundant by DESeq2 (in all of the pairwise comparisons between the group of interest and the other age-groups).

For each breed, Spearman’s rank test was used to find taxa associated with age and taxa positively/negatively correlated with *Campylobacter* relative abundance. Multiple testing correction with false discovery rate was performed and the correlation was considered significant if *p*_adj_ < 0.05. For *Campylobacter* correlation tests, only *Campylobacter*-positive animals were included in the analysis.

Core microbiome was identified for each breed at ASV and genus level and was defined as taxa detected in all age-groups with at least 10 reads within the group. To identify the shared and unique taxa among the age-groups, Venn diagrams were constructed with the online tool accessed through http://bioinformatics.psb.ugent.be/webtools/Venn/link.

For the study of broilers, all analyses were done both for the whole dataset to investigate the effect of age and/or diet, and in age-grouped subsets of data to study the effect of diet within each age-group. FRC data analyses were performed in the whole dataset to examine the effect of age. Significance (*p* < 0.05) was based on the corrected p-values.

## Results

### Sequencing output, preprocessing and taxonomic assignment

After the initial filtering and adaptors trimming process, samples from broilers accounted to a total of 9,887,438 paired-end reads with a median of 113,061 paired-end reads (IQR 95,211–137,688) per sample. Median length of the reads per sample was 246.1 bases (IQR: 244.5–247.1). After preprocessing, exclusion of ASVs classified as chloroplasts and mitochondria and removal of low-count ASVs as described in methods, the 8,879,083 amplicon reads from the 80 samples were classified into 1,163 ASVs. In free-range slow-growing chicken (FRC) samples, a total of 11,294,265 reads passed the filtering and adaptors trimming steps (median: 163,646; IQR: 116,527 – 211,636 per sample). Median length of the reads per sample was 247 bases (IQR: 246.5 – 247.4). Two samples were discarded (<50,000), leaving 10,041,046 amplicon reads from 68 samples classified into 2,033 ASVs. Number of reads that passed through each step of the DADA2 pipeline are presented in Supplementary Table S1.

Rarefaction curves generated from the ASVs (Supplementary Fig. S1) showed high sequencing coverage in all samples, and higher level of microbial diversity in FRC than in broilers. In both cases, chicken-to-chicken variation was observed in each age-group. Broilers fed different diets presented similar rarefaction curve patterns within each age-group (data not shown). In general, proportions of taxonomic assignation were similar in both of the breeds, but the number of unique taxa identified was notably higher in FRC (Supplementary Table S2).

### Alpha diversity

**Figure 1:**
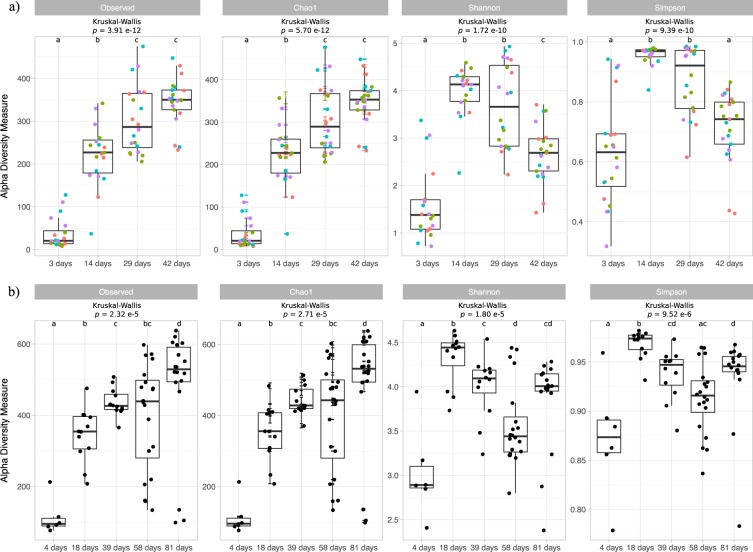
Boxplots representing alpha diversity metrics of richness (Observed ASVs and Chao1) and evenness (Shannon and Simpson) for samples from broilers (**a**) and free-range chickens (**b**) grouped according to age. Non-parametric Kruskal-Wallis test followed by post-hoc Wilcoxon rank sum test adjusted for multiple comparisons using the Benjamini–Hochberg method was conducted. Each point represents the diversity score for a sample; broiler chickens are color-coded according to diet (**a**). The box represents the first (Q1) and third (Q3) quartiles of the distribution, and the line within the box marks the median. The whiskers extend from Q1 and Q3 to the last data points within 1.5 × IQR and values beyond these whiskers are considered outliers. Boxplots not sharing a common letter above them are significantly different at *p*_adj_ < 0.05.

Alpha diversity indices of both datasets, broiler and FRC, showed similar pattern distribution with age (Fig. 1). A steady increase in species richness was observed as the chickens aged, as indicated by Observed ASV and Chao1. Both estimators showed similar patterns reassuring that the sequencing depth obtained was sufficient. Average Shannon and Simpson indices presented the lowest values in youngest chickens, indicating that the species present were not equally abundant, and reached the highest values at day 14 (broilers) and 18 (FRC), suggesting that the abundance of the different species was then more even. Both indices decreased gradually afterwards except in 81-day-old FRC, whose microbiome composition turned more evenly abundant again. Kruskal-Wallis tests of Richness, Shannon and Simpson indicated that bacterial diversity in chickens significantly differed from one age-group to another in both datasets. None of the alpha diversity indices were statistically different among diets in broilers, neither when the diet effect was analysed in the whole dataset nor restricting the dietary comparison to subsets of age-groups only and thereby controlling for the age effect. When comparing broilers and FRC, the latter showed higher values in all alpha diversity indices.

### Beta diversity

In both breeds, the overall microbial community structure exhibited clear and significant shifts by age (total variance explained R^2^ = 0.45, *p* < 0.001 in broilers and R^2^ = 0.44, *p* < 0.001 in FRC) based on PERMANOVA, and post-hoc analysis revealed significant differences between each possible pairwise comparisons between age-groups in both breeds.

**Figure 2:**
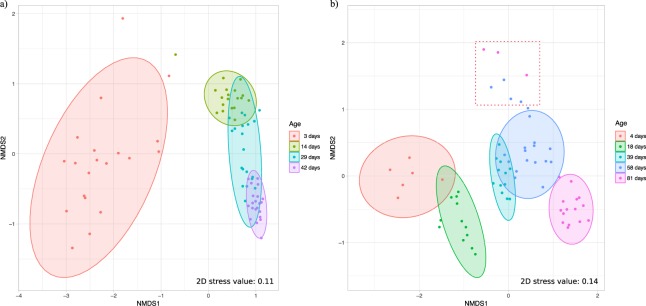
Non-metric multidimensional scaling (NMDS) plot based on Bray-Curtis dissimilarity matrix on relative abundance data in broilers (**a**) and free-range chickens (**b**). Colors indicate age groups. Ellipses indicate 95% confidence intervals of multivariate t-distribution around centroids of the groupings with age as factor. Dots within the square area in plot **b** represent chickens with typhlitis, which were excluded for the estimation of the ellipses (see text).

In broilers, betadisper revealed that individual variation in the community structure was signficantly greater in younger birds (3-day-old), while the lowest variance was found among 42-day-old broilers (F = 32.08, *p* < 0.001). Despite the lack of homogeneity in dispersion, the NMDS plot based on Bray-Curtis dissimilarity matrix still showed age-related clustering, with chickens of the same age clustering more closely together (Fig. 2a). Eleven of the 29-day-old broilers shared similar community structure with 14-day-old birds, while the remaining 9 chickens in the 29-day-old group were more similar in composition to 42-day-old broilers. In contrast, diet was not a significant factor neither for the whole dataset (R^2^ = 0.032, *p* = 0.718) nor when comparing different diets within age-groups (*p* > 0.05).

In FRC, betadisper indicated that microbiome community dispersion between individuals did not vary in the different age-groups (F = 1.74, *p* = 0.148), reinforcing the age effect in PERMANOVA results. The only exception were seven samples which were clearly distant from the centroids of its age-grouping. All these samples had in common the presence of hard caseous material in the lumen of the caeca, indicative of typhlitis. When these seven samples were excluded from their respective age-groups to calculate the 95% CI of the distribution around centroids of the ellipsoids, age clustering became tighter (Fig. 2b).

### Hierarchical clustering

**Figure 3:**
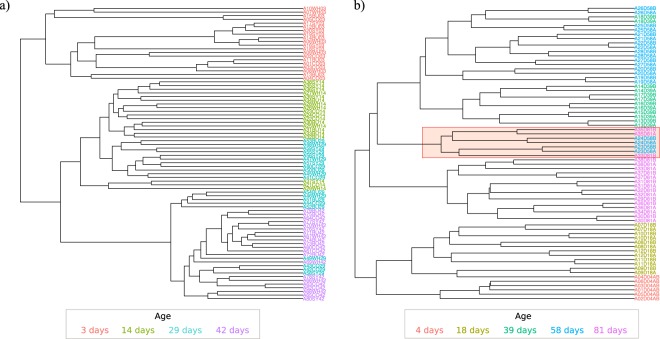
Hierarchical clustering analysis: Dendrogram of Bray-Curtis dissimilarity matrices between samples based on different age groups in broilers (**a**) and free-range chickens (**b**) with UPGMA method. The red frame in the free-range chickens dendogram (**b**) indicates the samples with typhlitis.

In agreement with the results observed in NMDS plots, dendogram of hierarchical clustering revealed that samples from 3, 14 and 42 day-old broilers (Fig. 3a) formed three separate clusters with samples from 14 day-old broilers clustering closer to 42 day-old than to 3 day-old broilers. On the other hand, samples from 29-day-old broilers grouped with both the 14-day-old and the 42-day-old broilers, with no clear predominant cluster defined. Again, no diet-related clustering was observed, neither in the whole dataset nor within subsets of age-groups. In FRC, pairs of caecal samples collected from the same chicken always clustered together. In general, chickens clearly clustered by age-group according to their bacterial community composition. The only exceptions were one 39-day-old chicken (both caeca) that were included in the group of 58-day-old chickens, and the seven samples from four chickens with signs of typhlitis. These seven samples (three from two 81-day-old chickens and four from two 58-day-old chickens) formed a different cluster in the dendogram independently of age (Fig. 3b).

### Microbial community dynamics

**Figure 4:**
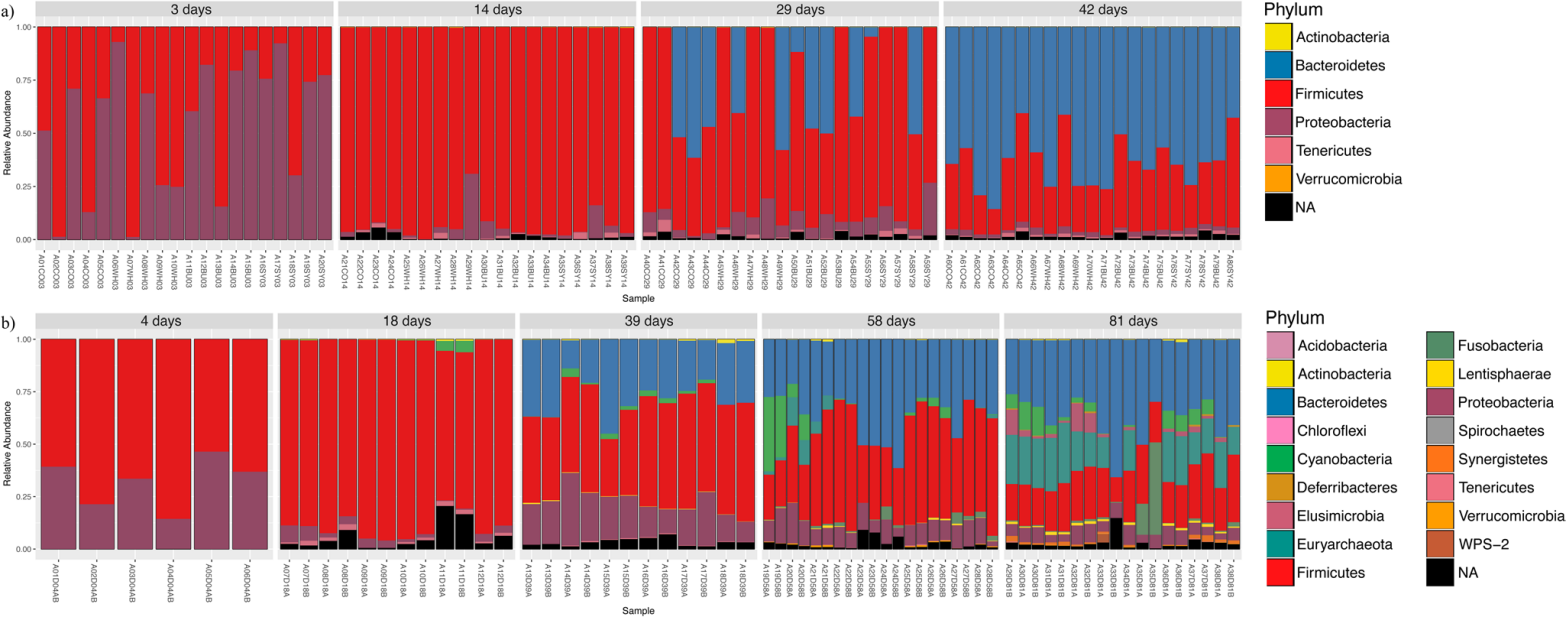
Microbial community composition of chicken caecal content. Stacked bar plots representing relative abundances of the different phyla in all samples in broilers (**a**) and free-range chickens (**b**).

**Figure 5:**
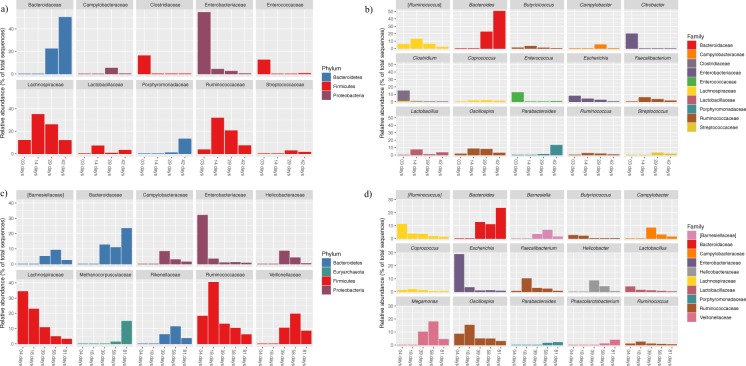
Relative abundance of the top 10 families and top 15 genera averaged over all samples for the age-groups for broilers (**a** and **b**) and free-range chickens (**c** and **d**).

**Figure 6:**
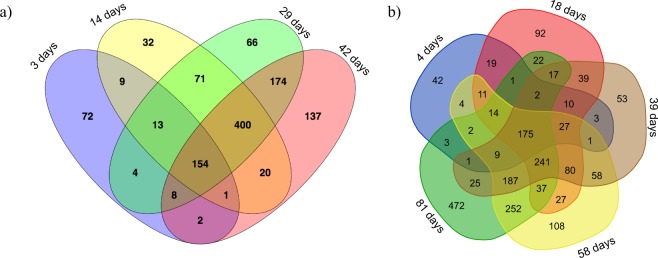
Venn diagramme illustrating core microbiome (ASVs) in broilers (**a**) and free-range chickens (**b**).

**Figure 7:**
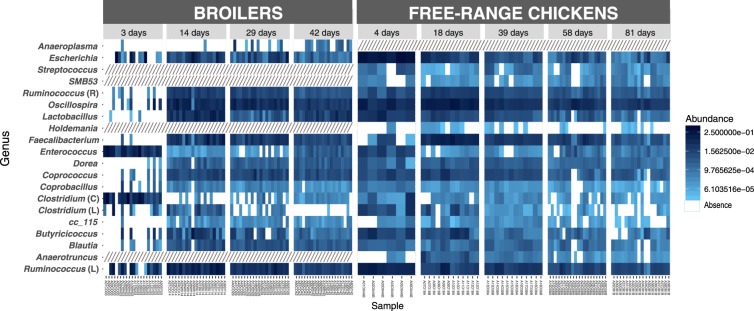
Heatmap representing classified genera within the core microbiome of each breed. Darker shades of blue represent higher relative abundance as indicated in the legend; white color represents absence. Boxes with diagonal pattern indicate that a particular genus was absent in at least one age group and therefore it was not considered as part of the core microbiome. Letter in brackets following a genus name indicates the family the genus belongs to (R – Ruminococcaceae, C – Clostridiaceae, L – Lachnospiraceae).

A complete list of all bacterial taxa (average relative abundance) identified for each age-group and breed in the caecal content samples is provided in Supplementary Table S3. In both breeds, microbial communities displayed different relative proportions according to age even at the phylum level, and microbial community underwent dramatic shifts along time (Figs 4 and 5). Microbial taxa consistently present over time (core microbiome) were represented by 154 ASVs within broilers and 175 ASVs within FRC (Fig. 6), and corresponded to 16 genera in broilers and 19 in FRC. Among them, 15 genera (mainly belonging to the order Clostridiales, phylum Firmicutes) were identified in every sampling point in both breeds with variations in relative abundance (Fig. 7).

Firmicutes and Proteobacteria together formed almost the entirety of the caecal microbiome of younger chickens, with Bacteroidetes reaching substantial levels in the second half of their lifespan. In both chicken breeds, Proteobacteria was a highly abundant phylum at first sampling, but proportions decreased thereafter. Thus, a 10-fold reduction in the relative abundance was observed on day 14 in broilers to 4.2% (from 54.7% on day 3) and on day 18 in FRC to 3.3% (from 31.9% on day 4). Proteobacteria shifted from a predominance of Enterobacteriales at younger ages, to Campylobacterales after the fourth week of age. Although Enterobacteriaceae was part of the core microbiome of both breeds, during the first two samplings it accounted for nearly all Proteobacteria (94–100%), but represented only 10% by the end of their productive period. Enterobacteriaceae in younger birds was mainly represented by *E*. *coli*, except in 3 day-old broilers in which *Citrobacter* accounted for 34% of Proteobacteria and was identified as a biomarker of that age-group (Table 1). Then, Campylobacterales became the main order within Proteobacteria. The highest abundance of *Campylobacter* was found in 29 day-old broilers (43% of Proteobacteria) and in 39 day-old FRC (67% of Proteobacteria). *Helicobacter* (45%) also accounted for the high abundance of Campylobacterales in 39 day-old FRC, but it was never found in broilers. At the end of their productive life, *Sutterella* (order Burkholderiales) became the most prevalent Proteobacteria genus in 42 day-old broilers (75% and biomarker of the group) and second in 81 day-old FRC (21%, after *Campylobacter* – 22%). In both breeds, the relative abundance of *Sutterella* was strongly correlated with time (spearman rho >0.75) (Supplementary Table S4) and with *Campylobacter* (rho = −0.61, *p* < 0.000 in broilers and rho = −0.42, *p* = 0.037 in FRC). At the end of the productive period, Enterobacteriaceae represented only about 10% of all Proteobacteria.

**Table 1:** Genera identified to be significantly representative of each age group in each breed as a result of combining LEfSe output and DESeq2 results. Genera were listed in descending order according to effect size in LEfSe.

Age	Genera
**Broiler**	
3 days	*Citrobacter*, *Enterococcus*, *Clostridium* (Clostridaceae)
14 days	*Anaerotruncus*, *Candidatus*_*Arthromitus*
29 days	*Campylobacter*
42 days	*Parabacteroides*, *Suterella*, *Dehalobacterium*
**Free-range chickens**	
4 days	*Clostridium* (Clostridaceae), *Epulopiscium*
18 days	*Oscillospira*, *Faecalibacterium*, *Anaerotruncus*, *Lachnospira*, *Ruminococcus* (Ruminococcaceae)
39 days	—
58 days	*Alistipes*, *AF12*, *Pseudoramibacter*_*Eubacterium*
81 days	*Elusimicrobium*, *Desulfovibrio*, *YRC22*, *Succinatimonas*, *Butyricimonas*, *Mucispirillum*, *Prevotella*, *Veillonella*, *Oxalobacter*

Firmicutes was dominated by families within the order Clostridiales (Lachnospiraceae, Ruminococcaceae and Clostridaceae) whose relative abundance varied with age and breed but formed part of the core microbiome of both chicken breeds. In fact, most genera identified in every sampling point in both chicken breeds belonged to the order Clostridiales (Fig. 7). Albeit present at important levels throughout the productive period in both breeds, highest proportions were detected in 14-day-old broilers (83% of bacteria) and 18-day-old FRC (81%). In 3-day-old broilers, this order was composed mainly by the family Clostridiaceae (50%), and Lachnospiraceae levels (37%) were three times higher than Ruminococcaceae (11%). Afterwards, Clostridiaceae family was almost depleted as Lachnospiraceae and Ruminococcaceae gained importance and the ratio between these two families became stable. Comparatively, levels of Clostridiaceae in FRC were never substantial (always below 3%). Instead, Lachnospiraceae and Ruminococcaceae were found in younger animals, along with Veillonellaceae after day 39. In both cases, the presence of Clostridiaceae became irrelevant in older individuals (<0.6%). *Clostridium* genus was present as core microbiome in both breeds, was a biomarker of the youngest birds (LDA effect size > 5) (Supplementary Table S5), and was negatively correlated with age, more strongly in FRC than in broilers (rho = −0.72 *vs*. −0.39) (Supplementary Table S4). However, in 3-day-old broilers, almost 70% of this genus was represented by *C*. *perfringens* while in FRC *C*. *perfringens* only represented 6%. LEfSe identified Lachnospiraceae as biomarker of 14 day-old broilers and 4 day-old FRC (LDA effect size > 6) (Supplementary Table S5). *Coprococcus* was among the top genera within Lachnospiraceae family in both broilers and FRC and it was present at all sampling points. The highest levels of Ruminococcaceae were observed in the second sampling point in both breeds. *Oscillospira*, *Faecalibacterium*, *Ruminococcus* and *Anaerotruncus* were among the top genera and they were observed consistently in both broilers and FRC throughout their lifespan. In fact, these four genera were identified as biomarkers of 18-day-old FRC, and *Anaerotroncus* was a biomarker of 14-day-old broilers (Table 1). Two families of the order Lactobacillales (Enterococcaceae and Lactobacillaceae) were also part of the core microbiome in both breeds. The genus *Enterococcus* (fam. Enterococcaceae) accounted for 12% of the bacteria in broilers, but represented only 2% of bacteria in FRC. Lactobacillaceae decreased with age in FRC from the highest levels found at 4 days, while it reached its highest peak of abundance in 14 day-old broilers due to the increase in *Lactobacillus* abundance. Opposite associations of *Lactobacillus* with *Campylobacter* were observed in broilers (rho = −0.43, *p* = 0.012) and FRC (rho = 0.29, *p* = 0.124). Another Lactobacillaceae genus, *Pediococcus*, was only found in the youngest chickens and it was 10 times more abundant in FRC than in broilers. Streptococcaceae was part of the core microbiome of FRC but it never represented more than 0.3% of the bacteria, whereas in 29 day-old broilers *Streptococcus* accounted for 3% of bacteria. At the end of their productive life, Firmicutes was still an important phylum in the caecal microbiota of both broilers (42 day-old) and FRC (81 day-old), but a clear phylum predominance shift from Firmicutes to Bacteroidetes was observed and Bacteroidetes became the main phylum (63.5% in broilers and 37.3% in FRC). In FRC, this reduction in the Firmicutes:Bacteroidetes ratio was lower than in broilers due to the presence of other phyla, in contrast to the much more limited community profile diversity found in broilers. *Parabacteroides* was identified as a potential biomarker of 42-day-old broilers whereas *Butyricimonas*, *YRC22* and *Prevotella* were biomarkers of 81-day-old FRC. In both breeds, *Parabacteroides* presented a strong correlation with age (spearman rho > 0.75)(Supplementary Table S4) and *Campylobacter* (rho = −0.59, p < 0.001 in broilers and rho = −0.39, *p* = 0.039).

Among the minor phyla, Tenericutes was present at steady levels throughout the productive period (0.002–1.5%), with *Anaeroplasma* as the main genus in broilers (Anaeroplasmataceae was a core family for broilers) and *Mollicutes* in FRC. Actinobacteria was always present except in 3-day-old broilers; levels ranged from 0.007–0.4%. In broilers, Actinobacteria was mainly composed of *Corynebacterium* and *Brachybacterium* and in FRC of *Corynebacterium*, *Bifidobacterium* and *Adlercreutzia*. Verrucomicrobia was only present in 42-day-old broilers and it was represented by only one genus, *Akkermansia*, at a very low proportion (0.0006%). In FRC, Verrucomicrobia was detected on days 58 and 81, and unassigned members of the family Cerasicoccaceae were more abundant than *Akkermansia*. A further 11 phyla were identified only in FRC, four of them reaching signifcant levels at the end of the productive period. Among these was the phylum Euryarchaeota (*vadinCA11* and unclassified reads from Methanocorpusculaceae family), the only archaea detected in this study, but present at high levels (17.1% in 81-day-old FRC). Also noteworthy was the presence of Cyanobacteria (4.3%), and Fusobacteriaceae (Fusobacteria) and Elusimicrobiaceae (Elusimicrobia) that reached 3.9% and 3.0%, respectively.

When chickens of both breeds were compared at the end of their productive life (42-day-old broilers *vs*. 81-day-old FRC), 30 common genera were identified, along with 35 genera exclusive of FRC and 7 only found in broilers (Table 2).

**Table 2:** Core genera between both breeds at the end of the productive period (42-day-old broilers and 81-day-old free-range chickens), and genera specific for each breed. Genera were sorted in alphabetical order.

Core Genera (n = 30)	Free-range chickens specific (n = 35)	Broilers specific (n = 7)
*Akkermansia*	*02d06*	*Anaeroplasma*
*Anaerofustis*	*Adlercreutzia*	*Anaerostipes*
*Anaerotruncus*	*AF12*	*Brachybacterium*
*Bacteroides*	*Alistipes*	*Ignatzschineria*
*Blautia*	*Anaerofilum*	*Jeotgalicoccus*
*Butyricicoccus*	*Avibacterium*	*Lachnospira*
*Campylobacter*	*Barnesiella*	*Staphylococcus*
*cc*_*115*	*Bifidobacterium*	
*Clostridium*	*Bilophila*	
*Coprobacillus*	*Butyricimonas*	
*Coprococcus*	*Candidatus Arthromitus*	
*Corynebacterium*	*Collinsella*	
*Dehalobacterium*	*Desulfovibrio*	
*Dorea*	*Dialister*	
*Enterococcus*	*Elusimicrobium*	
*Escherichia*	*Fusobacterium*	
[*Eubacterium*]	*Gallibacterium*	
*Faecalibacterium*	*Helicobacter*	
*Holdemania*	*Megamonas*	
*Lactobacillus*	*Mucispirillum*	
*Oscillospira*	*Odoribacter*	
*Parabacteroides*	*Oxalobacter*	
*Proteus*	*Paraprevotella*	
*Roseburia*	*Peptococcus*	
*Ruminococcus*	*Phascolarctobacterium*	
[*Ruminococcus*]	*Prevotella*	
*SMB53*	*Pseudoramibacter*_*Eubacterium*	
*Streptococcus*	*Rikenella*	
*Succinatimonas*	*Slackia*	
*Sutterella*	*Sphaerochaeta*	
	*Turicibacter*	
	*vadinCA11*	
	*Veillonella*	
	*Victivallis*	
	*YRC22*	

## Discussion

This study provided a thorough description of the microbiome in chicken caeca in two different breeds and management systems commonly used in the chicken meat industry, throughout their whole productive period in an attempt to provide a global picture of the microbial taxonomical diversity. For this purpose, high sequencing coverage of short amplicon reads was achieved by using targeted high-throughput sequencing technology. For more powerful and reproducible analyses, instead of using the threshold of >97% nucleotide sequence identity in the sequenced 16S rRNA gene region for operational taxonomic units (OTUs) definition, we identified sequence variants, ASVs, differing by as little as one nucleotide using the high-resolution DADA2 method, a model-based approach for correcting amplicon errors without constructing OTUs^47^. This method allows for more specific taxonomic assignation and permits future assignations when updated taxonomic information is available in the databases. By analysing biological sample replicates from each FRC (two ceca) we also demonstrated that both caeca had comparable microbiome composition, and that the analysis of a single caecum provides sufficiently representative data. This is in agreement with a previous study that analysed ten 42-day-old broilers from the same commercial flock^17^. Using these methods, broilers as a fast-growing breed raised in an intensive breeding system and a slow-growing breed of chicken grown in an extensive farming system with outdoor access were compared. Furthermore, the effect of feed supplementation with whey and calcium butyrate in broilers caecal microbiota was assessed to investigate if the beneficial effects on productive performance and duodenal histological integrity of dietary supplementation with these compounds^24^ were associated to diet-induced microbial shifts. Unexpectedly, our findings suggested that supplementation as described elsewhere^24^ did not alter the overall structure of the caecal microbiota of broilers. Other studies have shown that diet and feed additives^9,11,32,48^ are common factors that impact gut microbiome diversity, composition, and structure, but opposite results have also been reported^15,16,49^. In any case, since our analyses did not find any diet-associated changes, diet was henceforth excluded as a variable and results from broilers were directly compared with those from FRC.

In agreement with other studies^14,16,18,28,32^, the diversity and composition of caecal microbiome in both breeds were strongly influenced by age, increasing in complexity and biodiversity as the chickens grew. Breed was also an influential factor, with FRC presenting higher complexity in their caecal microbiome than broilers, consistent with results in a previous study^29^. Further supporting previous reports^10,17,48^, inter-individual variation was observed in the microbial community structure of birds of the same age, despite the fact that each type of chickens originated from the same breeder and flock and were reared under the same conditions. However, this variation was mainly due to differences in relative abundance rather than to taxonomic composition, which was consistent within each age-group and breed. In general, each age-group showed an age-associated community profile. Thus, three developmental stages of microbiota composition were identified in broilers during their lifespan and four in FRC, with an intermediate stage, as confirmed by UPGMA clustering and NMDS plots. The first stage, represented by 3-day-old broilers and 4 day-old FRC, showed a clearly immature microbiota dominated by Proteobacteria and Firmicutes. However, in broilers, Proteobacteria predominated (*Citrobacter* as main contributor) over Firmicutes (*Enterococcus*), and in FRC Firmicutes (*Ruminococcus*, fam. Lachnospiraceae) was more abundant than Proteobacteria (*E*. *coli*). In the second stage, represented by 14 day-old broilers and 18 day-old FRC, a drop in Proteobacteria proportion to less than 5% (*E*. *coli*) was observed resulting in an absolute dominance of the phylum Firmicutes (around 90%) represented by the families Lachnospiraceae and Ruminococcaceae, at similar levels in broilers but enriched in Ruminococcaceae over Lachnospiraceae in FRC. The relatively high abundance of members of the families Lachnospiraceae (*Coprococcus*, *Roseburia*, *Anaerostipes*) and Ruminococcaceae (*Faecalibacterium*, *Anaerotruncus*), which have been shown to express enzymes favoring the production of butyrate over propionate^50,51^, is likely associated to the high nutrient requirements for growth during this phase since butyrate is the most preferred source of energy in quickly growing chickens^52^. The third stage would be represented by 42 day-old broilers and 58 day-old FRC, where a replacement of members of Firmicutes with Bacteroidetes occurred, in a more gradual manner in FRC than in broilers. Firmicutes was still represented by Lachnospiraceae and Ruminococcaceae in broilers, whereas in FRC Veillonellaceae accounted for almost half of the Firmicutes, with *Megamonas* as the most abundant genus. Within the phylum Bacteroidetes, *Bacteroides* clearly prevailed in broilers (50.3%), but represented a smaller porportion in FRC (10.8%) along with members of Rikenellaceae (11.3%) and Barnesiellaceae (9.1%). Remarkably, *Megamonas* and *Bacteroides* produce propionate as the main end product of the degradation of complex plant polysaccharides^51,53^. Although propionate is a less preferred energy source than butyrate for rapid growth, at this stage, its production as the result of digestion of complex polysaccharides might represent an efficient balance between energy acquisition from available nutrients and sustained growth^51^. The very rich and diverse caecal microbiome of 81 day-old FRC would constitute stage 4, represented by 14 phyla. The most prevalent phyla were Bacteroidetes, Firmicutes and Euryarchaeota, with Bacteroidaceae, Methanocorpusculaceae and Veillonellaceae as the most representative families, respectively. The microbial community of broilers aged 29 days and FRC aged 39 days could be considered a transition between stages 2 and 3, when some of the birds still presented similar microbiota composition to the previous age-group while others were already diverging towards a more mature microbiota structure. This situation was described elsewhere when comparing 14-day-old and 28-day-old broilers^14^.

Microbiota development is probably a continuum process of microbial communities succession, where certain taxa are replaced by others as chickens grow. Based on the time points assessed, the stages described here for the whole chicken lifespan clearly showed the evolution from an early immature stage to a mature microbiota that differed between breeds. In agreement with other reports on broiler caecum microbiota, early stages were dominated by Proteobacteria (at the expense of Enterobacteriaceae), Firmicutes increased in later phases^28,30,32^ and Bacteroidetes was enriched at the end of the productive period^21^. However, studies on the microbial community in the caecum of meat chickens older than 42 days are scarce^29^. Therefore, we compared the results of FRC with those reported for laying hens^18^, and found some similarities for stages 1–3. However, higher microbial diversity, particularly in the second half of the productive period, was observed in this study, and higher levels of Bacteroidaceae and lower proportions of Porphyromonadaceae were found within the Bacteroidetes. The outdoor access and the consumption of grass might be the explanation for this diverse and complex microbiota. Early microbial exposure has been described to play a major role in determining the distinctive characteristics of the microbial community^8^. In fact, the microbial community structure of FRC was notably distinct before and after outdoor access, when grass was introduced in the diet. Twelve days after outdoor grazing, five new classes emerged (Deltaproteobacteria, Epsilonproteobacteria, RF3, Fusobacteriia, and Lentisphaeria), the last two never found in broilers. In the second half of the productive period, the number of different phyla that reached important levels was far larger in FRC than in broilers, and included members of Euryarchaeota, the only archaeal phylum detected in this study, and only in FRC. Here, this methanogenic archaea (unclassified reads of the Methanocorpusculaceae family and *vadinCA11*) accounted for 17% of the total microbial community of 81 day-old FRC. Previous studies had already described the presence of methanogenic archaea in chickens caeca^29,54,55^ but always at lower abundances than those found here. Methanogens have been described to act as major consumers of the hydrogen accumulated from bacterial fermentation that leads to reduced or less energy-efficient fermentation^56^. By removing hydrogen, methanogenic archaea contribute to increase microbial fermentation rates and enhance host energy capture^57^.

Interestingly, the above mentioned classification into different developmental stages of microbiota composition had some exceptions. Several FRC which clearly separated from their respective age-group turned out to show signs of typhlitis in the lumen of their caeca. Microbial communities of these individuals were less diverse and more similar to each other than to other members of their respective age-groups. Studies in animals have shown that alterations in the normal physiology of the gut can decrease/alter microbal community diversity^58–60^. Further analyses are needed to define taxa related to this dysbiosis, but these results showed that dysbiosis can be detected by microbial community profile investigation.

Despite differences associated to age and production type or breed, there was a core microbiome for both chicken breeds which was mainly composed by members of the Firmicutes phylum. In fact, the only phyla present during the whole productive lifespan of both broilers and FRC were Firmicutes and Proteobacteria. These two phyla accounted for almost the total caecal microbiome of younger chickens, and Bacteroidetes only reached important levels in the second half of their lifespan. Although this trend was common to both breeds, relative abundances were different since the composition of caecal microbiota was richer and more complex in FRC than in broilers and a higher number of taxa was always observed. Species found in FRC but not in broilers included propionate producers like *Megamonas*, methanogenic archaea of the phylum Euryarchaeota or sulfate reducers like Desulfovibrionaceae. Besides, some taxa that have been associated with healthy gut were present exclusively or at higher levels in FRC. This was the case of *Bifidobacterium*, a genus of the phylum Actinobacteria that produces lactic acid as a major product of glucose fermentation and elicits a beneficial effect on the host intestinal ecosystem^61^. Actinobacteria is a phylum commonly found in the avian gastrointestinal tract^62^, and plays a crucial role in the development and maintenance of intestinal homeostasis^61^. Here, as described in other studies^17,21,29,30^, levels of Actinobacteria in the caeca of both chicken breeds were low, but abundance and diversity of the representing taxa were always higher in FRC than in broilers. Among the minor phyla associated to the mature microbiota, it was noteworthy the presence of bacteria with potential to stimulate mucus layer formation and therefore associated with healthy gut^63,64^, like *Akkermansia* (phylum Verrucomicrobia) found at substantially higher levels in FRC than broilers, and *Mucispirillum* (phylum Deferribacteres) found only in FRC and at higher levels in 81-day-old FRC where it was a biomarker. Although further studies are needed to fully understand the role of gut microbiota in defining states of health and disease, available data suggest that richer and more complex microbial communities with a high level of functional redundancy could confer advantages under environmental changes and as thus be considered more robust^6–8^.

Considering the major role of poultry in human campylobacteriosis, associations of *Campylobacter* relative abundance with the different age-groups or other taxa were investigated. When *Campylobacter* (mainly *C*. *jejuni* and/or *C*. *coli*) enters the flock, infection spreads rapidly and colonizes the caeca of most birds at high levels, and birds remain infected throughout their productive life^25^. Here, *Campylobacter* was identified at differentially higher abundance levels in 29 day-old broilers and 39 day-old FRC (LDA effect size > 5). This was expected for 29 day-old broilers, which had been experimentally inoculated with *Campylobacter* at 15 days of age^24^, but a high abundance of *Campylobacter* was also found in 39 day-old FRC as a consequence of a natural infection. This confirms the widespread of this genus in chicken poultry production, particularly in extensive systems where strict biosecurity measures are difficult to implement. At the end of their productive life, when Enterobacteriaceae represented only about 10% of all Proteobacteria, *Sutterella* (order Burkholderiales) became the most prevalent Proteobacteria genus in 42 day-old broilers and second in 81 day-old FRC. Spearman correlation analysis showed a negative correlation of *Sutterella* with *Campylobacter* in both breeds. *Sutterella* has been identified in humans, dogs and turkey, and it has been proposed as a direct competitor for *Campylobacter* in the intestinal ecosystem^65^ and associated to low feed conversion rate broilers^66^ and high weight rabbits^67^. Another genus negatively associated with *Campylobacter* in both breeds was *Parabacteroides* (phylum Bacteroidetes). This genus has been associated with healthy gut microbiota and certain species have been suggested to exert positive effects in the host immunity^68^. On the other hand, several genera of the phylum Firmicutes (*Faecalibacterium*, *Anaerotruncus*, *Blautia*) were positively associated with *Campylobacter*. In broilers, Lactobacillaceae showed a considerable decrease in relative abundance in 29 day-old broilers, when *Campylobacter* load was the highest as determined by real-time PCR elsewhere^24^, and *Lactobacillus* abundance was negatively correlated with *Campylobacter* as confirmed here by 16S rRNA NGS. This is in agreement with studies that showed a decrease in Lactobacillaceae abundance associated to increased levels of *Campylobacter*^69^. However, in the case of FRC, the correlation between *Lactobacillus* and *Campylobacter* was positive but weaker and statistically non-significant. Given the association between the consumption of *Campylobacter*-infected chickens and human campylobacteriosis, these associations and the possible influence of microbial community composition on *Campylobacter* levels are of great interest and require further studies.

In conclusion, age was the strongest influencing factor in caecal microbiota composition of chicken, and, in general, each age-group showed an age-associated community profile, with a transition period at the middle of their lifespan. However, there were substantial differences in the phylogenetic composition of caecal microbiota between broilers and FRC, microbiota being richer and more complex in FRC, which had access to grass and soil outside, than in broilers reared intensively on a more limited diet. Broilers are under higher presure than FRC to grow rapidly and need a microbial community that provides optimal recovery of energy from food, whereas FRC grow slowly and have contact with complex microbial ecosystems in the environment. The higher diversity in microbial community and the presence exclusively or at higher levels in FRC of some taxa that have been associated with healthy gut, suggested that FRC harboured a more healthy microbiota. Finally, by microbial community comparison we were able to identify a group of animals with dysbiosis, *i.e.*, a group of FRC with signs of typhlitis in the lumen of their caeca which stood out after showing a microbial community less diverse and more similar to each other than to other members of their respective age-groups.

## Supplementary information

Supplementary Tables and Figures

Supplementary Table S3

Supplementary Table S5

## Data Availability

The raw paired-end reads of the 16S rRNA gene sequencing data were deposited in the European Nucleotide Archive (ENA) database under the accession number PRJEB29068.
