# Supplementary material for: 16S rRNA amplicon sequencing characterization of caecal microbiome composition of broilers and free-range slow-growing chickens throughout their productive lifespan
Source: Sci Rep. 2019 Feb 21;9:2506. doi: 10.1038/s41598-019-39323-x (PMC6385345; doi:10.1038/s41598-019-39323-x)
Supplement: Supplementary file 1 — Supplementary Tables and Figures [file 41598_2019_39323_MOESM1_ESM.docx]

**16S rRNA amplicon sequencing characterization of caecal microbiome composition of broilers and free-range slow-growing chickens throughout their productive lifespan**

Medelin Ocejo^1^, Beatriz Oporto^1^ and Ana Hurtado^1,*^

^1^NEIKER-Instituto Vasco de Investigación y Desarrollo Agrario, Animal Health Department, Derio, 48160, Spain

*ahurtado@neiker.eus

**Supplementary Tables and Figures**

**Supplementary Table S1. Number of reads that passed through each step of the pipeline in DADA2 for broilers and free-range chickens datasets.**

| **Age** | **n ª** | **Input** | | | | **Filtered** | | | **Denoised** | | | | **Merged** | | | | **Tabled** | | | | **Non-chimeric** | | | | | **Clean** | | | |  |
| --- | --- | --- | --- | --- | --- | --- | --- | --- | --- | --- | --- | --- | --- | --- | --- | --- | --- | --- | --- | --- | --- | --- | --- | --- | --- | --- | --- | --- | --- | --- |
|  |  | **Mean** | | **SD** | **Mean** | | **SD** | | **Mean** | | **SD** | | **Mean** | | **SD** | | **Mean** | | **SD** | | **Mean** | | **SD** | | **Mean** | | | **SD** | |  |
| **Broilers** |  |  | |  |  | |  | |  | |  | |  | |  | |  | |  | |  | |  | |  | | |  | |  |
| 3 days | 20 | 116868.3 | | 42723.4 | 108785.6 | | 39026.3 | | 108785.6 | | 39026.3 | | 108750.6 | | 39020.9 | | 108750.4 | | 39021.1 | | 108373.4 | | 39113.9 | | 108367.9 | | | 39115.4 | |  |
| 14 days | 19 | 111009.8 | | 45546.4 | 98606.4 | | 43441.7 | | 98606.4 | | 43441.7 | | 98493.7 | | 43413.2 | | 98412.8 | | 43418.7 | | 97869.6 | | 43362.7 | | 97865.3 | | | 43362.3 | |  |
| 29 days | 20 | 140027.8 | | 64832.3 | 126423.3 | | 60732.0 | | 126423.3 | | 60732.0 | | 126221.5 | | 60713.0 | | 126219.4 | | 60715.1 | | 125747.3 | | 60443.0 | | 125735.8 | | | 60445.2 | |  |
| 42 days | 21 | 123647.4 | | 46032.9 | 111836.6 | | 42103.4 | | 111836.6 | | 42103.4 | | 111592.0 | | 42104.5 | | 111592.0 | | 42104.5 | | 111334.0 | | 42066.2 | | 111312.8 | | | 42059.3 | |  |
| **Free-range chickens** | | | | | | | | | | | | | | | | | | | | | | | | | | | | | |  |
| 4 days | 6 | 119751.3 | 42305.2 | | | 108301.0 | | 38593.1 | | 108301.0 | | 38593.1 | | 108130.2 | | 38572.1 | | 108099.8 | | 38544.7 | | 107964.8 | | 38443.3 | | | 107952.5 | | 38446.0 | |
| 18 days | 12 | 153290.8 | 54570.6 | | | 139436.8 | | 50398.4 | | 139436.8 | | 50398.4 | | 139050.3 | | 50267.9 | | 139032.1 | | 50256.5 | | 138238.7 | | 50263.9 | | | 138218.0 | | 50262.6 | |
| 39 days | 12 | 145371.4 | 52813.1 | | | 133643.9 | | 48039.7 | | 133643.9 | | 48039.7 | | 133207.9 | | 47940.1 | | 133152.3 | | 47896.6 | | 132080.6 | | 47477.9 | | | 131941.1 | | 47454.9 | |
| 58 days | 20 | 168552.9 | 54017.5 | | | 153331.5 | | 48464.3 | | 153331.5 | | 48464.3 | | 152948.4 | | 48398.6 | | 152897.7 | | 48370.9 | | 152371.6 | | 48226.5 | | | 152269.1 | | 48206.1 | |
| 81 days | 20 | 180774.5 | 63566.0 | | | 160104.5 | | 56730.2 | | 160104.5 | | 56730.2 | | 159602.0 | | 56616.2 | | 159587.5 | | 56609.4 | | 159186.5 | | 56513.9 | | | 172557.7^b^ | | 40646.5 | |

ª n, number of samples. In the case of broilers and 4-day-old free-range chickens, the number of samples equals the number of animals analysed; in the case of free-range chickens of 18, 39, 58 and 81 days of age, where each of the two caeca of each animal were individually analysed, the number of samples doubles the number of animals analysed.

^b^ Two samples (caeca from two different animals) were discarded due to low number of reads (<50,000) after the different filtering steps, so that final clean reads corresponded to 18 samples.

| **Taxonomic level** | **% taxonomic assignment**  **(n unique taxa)** | |
| --- | --- | --- |
|  | **Broilers** | **Free-range chickens** |
|  | **(1163 ASVs)** | **(2033 ASVs)** |
| Kingdom | 100.0 (1) | 100.0 (2) |
| Phylum | 97.5 (6) | 95.7 (17) |
| Class | 96.6 (14) | 94.2 (28) |
| Order | 96.2 (20) | 93.5 (37) |
| Family | 63.9 (32) | 64.6 (55) |
| Genus | 36.3 (43) | 35.2 (77) |
| Species | 5.8 (19) | 5.9 (38) |
|  |  |  |

**Supplementary Table S2. Proportions of taxonomic assignment of the amplicon sequence variants (ASVs)** **and number of unique taxa per taxonomic rank in both breeds.**

**Supplementary Table S4. Spearman’s rank correlations between age (days) and relative abundance of genera in caecal microbiota of broilers and free-range chickens.**

| **Genus relative abundance**^a^ | **Age (days)** | | | |
| --- | --- | --- | --- | --- |
|  | **Broilers** | | **Free-range chickens** | |
|  | **rho (ρ)^b^** | ***p*_adj_^c^** | **rho (ρ)^b^** | ***p*_adj_^c^** |
| *Bacteroides* | **0.88** | **0.000** | 0.75 | **0.000** |
| *SMB53* | 0.81 | **0.000** | 0.30 | **0.018** |
| *Streptococcus* | 0.79 | **0.000** | 0.05 | 0.731 |
| *Dehalobacterium* | **0.78** | **0.000** | 0.22 | 0.083 |
| *Sutterella*^d^ | **0.78** | **0.000** | **0.85** | **0.000** |
| *Parabacteroides*^d^ | **0.75** | **0.000** | **0.84** | **0.000** |
| *Campylobacter* | 0.71 | **0.000** | 0.39 | **0.002** |
| *Dorea* | 0.69 | **0.000** | **-0.46** | **0.000** |
| *Anaerostipes* | **0.67** | **0.000** | -0.30 | **0.018** |
| *Corynebacterium* | **0.65** | **0.000** | 0.13 | 0.338 |
| *Anaeroplasma* | 0.51 | **0.000** | -0.53 | **0.000** |
| *Coprococcus* | 0.48 | **0.000** | -0.64 | **0.000** |
| *cc_115* | 0.48 | **0.000** | -0.48 | **0.000** |
| *Lactobacillus* | 0.47 | **0.000** | **-0.76** | **0.000** |
| *Blautia* | 0.46 | **0.000** | -0.46 | **0.000** |
| *Roseburia* | 0.45 | **0.000** | 0.01 | 0.921 |
| *Anaerofustis* | 0.39 | **0.001** | -0.05 | 0.734 |
| *Faecalibacterium* | 0.39 | **0.001** | -0.48 | **0.000** |
| *Anaerotruncus* | 0.33 | **0.005** | -0.48 | **0.000** |
| *Proteus* | 0.32 | **0.006** | -0.37 | **0.003** |
| *Staphylococcus* | 0.28 | **0.019** | -0.11 | 0.403 |
| *Oscillospira* | 0.24 | 0.051 | -0.65 | **0.000** |
| *Lachnospira* | 0.22 | 0.062 | -0.39 | **0.002** |
| *Butyricicoccus* | 0.15 | 0.213 | -0.61 | **0.000** |
| *Succinatimonas* | 0.15 | 0.213 | **0.72** | **0.000** |
| *Akkermansia* | 0.15 | 0.213 | **0.37** | **0.003** |
| *Coprobacillus* | 0.07 | 0.554 | **-0.65** | **0.000** |
| *Escherichia* | 0.02 | 0.835 | -0.67 | **0.000** |
| *Candidatus_Arthromitus* | -0.09 | 0.465 | -0.44 | **0.000** |
| *Ruminococcus* (Lachnospiraceae) | -0.16 | 0.173 | **-0.49** | **0.000** |
| *Ruminococcus* (Ruminococcaceae) | -0.16 | 0.176 | -0.68 | **0.000** |
| *Enterococcus* | -0.29 | **0.013** | 0.22 | 0.092 |
| *Pediococcus*^d^ | **-0.31** | **0.009** | **-0.35** | **0.005** |
| *Epulopiscium*^d^ | **-0.35** | **0.003** | **-0.41** | **0.001** |
| *Clostridium* (Lachnospiraceae)^d^ | **-0.39** | **0.000** | **-0.72** | **0.000** |
| *Clostridium* (Erysipelotrichaceae) | **-0.44** | **0.000** | -0.20 | 0.125 |

1. Genera are listed in descending order according to the Spearman´s correlation coefficient in broilers.
2. Spearman´s correlation coefficient. Positive values indicate positive association between age and genus relative abundance while negative values express negative relationships. Values in bold indicate those associations in which the increase/decrease trend over time was continuous.
3. *p*-value of Spearman’s rank correlation between age (days) and genus level composition of caecal microbiota after FDR correction. Significant p_adj_-values are in bold.
4. Genera in which the correlation resulted significant and the increase/decrease trend over time was continuous in both breeds.

Attached separately as Excel files in electronic supplementary material:

**Supplementary Table S3. Average relative abundance of taxa in broilers and free-range chickens for each age group, with ASVs collapsed to species rank.** Relative abundance values >2% are highlighted in red.

**Supplementary Table S5. Differential analysis results of differentially abundant taxa (DESeq2) and potential biomarkers of age groups (LEfSe) for broilers and for free-range chickens.**

Tabs with “DESeq2” prefix:

Differentially abundant taxa as calculated by DESeq2 across age groups by chicken breed. Taxa were agglomerated to genus level for each breed. Values represented log2 fold change in average relative abundance of the latter group compared to the first group in comparison, and significant values (padj < 0.05) were formatted in red and bold font. Coloured bars represent positive (blue) and negative (red) values of the fold change.

Tabs with “LEfSe” prefix:

Important taxa of each age group at each taxonomic rank as identified by LEfSe in broiler and free-range chickens. Only those classified taxas with LDA score > 2 are shown. Taxa are listed in descending order according to their effect size within each age group. Adjusted p-value cutoff = 0.05.

**Supplementary Figure S1. Rarefaction curves plotting the number of observed ASVs over the number of sequencing reads sampled per chicken from the broilers (A) and free-range chickens (B) datasets.**
